# A Study on Health Seeking Behaviors of Patients of Post-Kala-Azar Dermal Leishmaniasis

**DOI:** 10.1155/2015/314543

**Published:** 2015-12-15

**Authors:** Ariful Basher, Proggananda Nath, Shah Golam Nabi, Shahjada Selim, Md Fashiur Rahman, Satya Ranjan Sutradhar, Abul Faiz, Matiur Rahman Bhuiyan, Be-Nazir Ahmed, Ridwanur Rahman

**Affiliations:** ^1^Department of Infectious & Tropical Diseases, Mymensingh Medical College Hospital, Mymensingh, Bangladesh; ^2^Centre for Medical Education, Dhaka, Bangladesh; ^3^Department of Endocrinology, Bangobandu Sheikh Mujib Medical University, Dhaka, Bangladesh; ^4^Mymensingh Medical College Hospital, Mymensingh, Bangladesh; ^5^Department of Medicine, Mymensingh Medical College, Mymensingh, Bangladesh; ^6^Dev Care Foundation, Dhaka, Bangladesh; ^7^Department of Pathology, Mymensingh Medical College Hospital, Mymensingh, Bangladesh; ^8^Department of Microbiology, National Institute of Preventive & Social Medicine (NIPSOM), Dhaka, Bangladesh; ^9^Department of Medicine, Shaheed Suhrawardy Medical College, Dhaka, Bangladesh

## Abstract

Post-Kala-Azar Dermal Leishmaniasis (PKDL) remains a major public health threat in Bangladesh. A cross-sectional study was carried out in Surya Kanta Kala azar Research Centre (SKKRC), Mymensingh, from January 2012 to July 2013 to evaluate the health seeking behaviour and the length of delay of PKDL management. The consecutive 200 diagnosed PKDL cases that got treatment in SKKRC hospital were subjected to evaluation. Most (98%) of the patients were not aware and had no knowledge about PKDL, though 87.5% had a history of history of Kala-azar treatment. Many patients reported first to village doctor (15.5%), the pharmacy shop (10%), or traditional health provider (7.5%) upon recognition of symptom. The time between the initial symptom recognition and first medical consultation (patient delay) ranged from 10 days to 4745 days (13 years) with a median of 373 days (mean: 696; IQR: 138 to 900 days). The time between first medical consultations to definite treatment (system delay) ranged from 0 days to 1971 days (5.4 years), with a median delay of 14 days (mean: 46.48; IQR: 7 to 44 days) that was reported in this study. Age, education, occupation, and residential status had significant association with patient delay (*P* < 0.05). Educational status, occupation, number of treatment providers, and first health care provider had a significant association with system delay (*P* < 0.05). Success in PKDL diagnosis and treatment requires specific behavior from patients and health care providers which facilitate those practices.

## 1. Introduction

Bangladesh is one of the five countries bearing the major (90%) global burden of visceral leishmaniasis (VL), caused by parasite Leishmania donovani [1]. Twenty million people (18% of the total population) are considered to be at risk for VL and 1000–2000 new cases in Bangladesh are annually identified [1, 2]. Post-Kala-Azar Dermal Leishmaniasis (PKDL), a curious phenomenon believed to be developed after treatment of Kala-Azar [3], presents with wide varieties of skin lesions ranging from hypopigmented marks, to erythematous papules, nodules, and others that appear in individuals. Unlike Kala-Azar, which is ultimately fatal without treatment, it is not usually associated with systemic illness, and patients can remain infectious for years or even decades and it serves as potential reservoir for anthropometric leishmanial transmission [4].

PKDL usually occurs among the poorest segments of rural population. Few data exist on social behavior and economic consequence of PKDL patients. Delay in diagnosis and treatment increases the risk of transmissibility and morbidity [5, 6]. This is true that sociocultural aspects of care or health seeking behavior particular to Bangladesh are unknown. As humans are the only hosts in Southeast Asia, treatment delay may increase the risk of reservoir for infection. Different social and community factors may play an important role in determining care seeking behavior and the ability to control the disease. Little is known about how individual and community health professionals perceive the disease of PKDL. However, such information is vital because successful control program requires a high level of understanding the pattern of health care seeking behavior.

Evidences revealed that health system, community, family, and other personal issues are influencing factors for the effective health seeking behaviors and case findings [7]. The characteristics of affected patients who reside in endemic area may lead to earlier diagnosis and treatment and thus improve the outcome. The study was designed to investigate PKDL patient care-seeking behavior and the causes of delay of treatment on affected household and identify the risk factors associated with long delay, if any in particular is related to choice of initial health care provider. Delay in receiving treatment for PKDL could be divided into “patient delay” (time from symptoms recognition to initial medical consultation) and “system delay” (time from first medical consultation to definite treatment) [8, 9]. Paucity of any evidence on the magnitudes of delay components (patient delay, health service delay, and total delay) and the factors associated with delay in care- seeking behavior among PKDL patients were among the reasons to conduct this study.

## 2. Research Design and Methods

### 2.1. Study Design

This was a cross-sectional study conducted from January 2012 to July 2013. The patient who had confirmed diagnosis of PKDL and got treatment in (Suryia Kanta Kala-Azar Research Centre) SKKRC in Mymensingh was selected successively and interviewed. The pretested questionnaires were used to collect information from the patients. Sample size was determined with a single population proportion formula. Based on the estimated incidence of PKDL (21 cases per 10,000 person-years in 2007), the rising PKDL of the countries studied, a maximum allowed error of 10%, and a 95% confidence interval, the least reliable sample size would have been 150 patients. However, as it was planned to perform bivariate logistic regression analysis, the rule of thumb was used; hence the sample size was calculated at 200 study subjects owing to the large number of studied variables. Logical cross tabulations were analyzed along with Chi-Square test in SPSS 14. Standardized definitions of the different types of delay were followed. Both descriptive and inferential statistics were determined. The rate of presentation to health facility or start of treatment was calculated as Rate = *e*/*d*, where *e* is the number of events occurring over time (such as presentation to health institution or commencement of treatment) and *d* is the total number of person days (see Table 1 for the specific calculations). The level of significance was set at *P* < 0.05. Frequencies and proportions were used for the descriptive analysis. Differences in proportions were compared for significance using Chi-Square (*χ* ^2^) test. The ANOVA test or 2-sample student's *t*-test and *χ* ^2^ test were used to assess differences considering each group is approximately normal. Cross-tabulations and bivariate logistic regression were done to identify the most important predictor variables of PKDL treatment seeking behavior.

### 2.2. Patient Delay

Time between the initial symptoms recognition by the patient and first medical consultation.

### 2.3. System Delay

Time between first medical consultations to definite treatment.

### 2.4. Total Delay

Time from the onset of symptoms until treatment was commenced and was made of above 2 components.

### 2.5. Focus Group Discussion

Quick participatory methods (Focus Group Discussion) were used to generate information on the myths, knowledge, attitudes, practices, and perceptions of the patients about Post Kala-Azar Dermal Leishmaniasis.

Each FGD involved 10 participants including equal number of both sexes and age ranged from 18 to 60 years. Total two focus group discussions were held in the study area. The informant groups were asked to discuss PKDL, especially the local terminology, knowledge, belief, attitude, and practice about PKDL.

Ethical clearance was obtained from Mymensingh Medical College Hospital and partially funded by DGHS (Director General of Health Services), Bangladesh. After giving all this information, signed written informed consent was obtained from the patient/guardian by signature/finger impressions. In case of subjects below 18 years, assent was taken.

## 3. Result

### 3.1. General Characteristics of the Patients

The patient's age ranged between 4 years and 70 years (mean: 24.4 years). Mean BMI was 20.6 ± 4.05 kg. The average monthly family cash income less than 100 US$ (1 US$ = 80 taka) was 76%, whereas only 20% had income >US $150.00/month. Some characteristics of the study participants are shown in Table 1. The median travel time to the nearest public health facility in a single trip was 1 hr (inter quartile range [IQR] = 1.5 hours) and 0.33 hours (IQR = 0.25 hours) for rural and urban patients, respectively, with a statistically significant difference (Mann-Whitney *U* test = 7.97, *P* < 0.001).

### 3.2. Clinical Characteristics with Background History

Eighty-seven percent (87%) had history of visceral leishmaniasis (VL) with the median number of years of PKDL presentation and previous treatment for VL was 4.5 years, ranging from 1 to 26 years. Half of the cases (50.6%) were presented in 5 years of VL treatment.

Most of the cases were treated with Sodium Stibogluconate (SSG) for VL (70.5%). Of these, 1/3 had received a total dose that was lower than recommended by the World Health Organisation (28–30 days of Sodium Stibogluconate (SSG)). Two patients presented after receiving liposomal amphotericin (total 15 mg/kg body weight). Four patients had history of both SSG and miltefosine (2.5 mg/kg for 28 days) treatment for VL in different time period. Thirty-eight percent had family history of VL.

Four patients presented after having received a previous treatment for PKDL with SSG (120 doses). All of those previously treated patients reported to have been cured before it reappeared again. It remains unclear whether those patients were labelled as a relapse or can be considered new case. Four patients were treated with miltefosine for 12 weeks and 3 patients got liposomal amphotericin 5 mg/kg body weight for 6 days without any improvement even after one year.

Symptoms and signs of PKDL were experienced by the patients including hypomelanotic, macular, papular, or nodular rashes (Table 2). Most of the patients (74%) presented with hypomelanotic spot amongst 78.5% considered grade III involving abdomen and limbs [10]. Diagnosis was mainly based on using rapid diagnostic test (RK-39) and skin manifestation. Ninety-seven percent were positive RK-39 tests and slit skin biopsy among 75 cases (out of 140 cases). Significant difference was not observed in time to presentation or initiation of treatment with all the symptoms after examining the symptoms separately or in groups.

### 3.3. Knowledge and Practice about the Disease

This study revealed that only 7% had heard the name of PKDL (Table 3). Almost all the respondents (98%) were not aware and had no knowledge about the diseases, though 87.5% had history of VL treatment. However 21% believed that the disease is possibly curable. Most of the patients (90%) learned about the disease from hospital. Among the respondents, 70% always used mosquitoes net at bed time but 28% regularly used to sleep at day time. Almost all (96.5%) had sleeping bed. Insecticide spray was done among 87% respondents house in the last year (Table 3). Only nine percent (9%) of the respondents believed that Kala-Azar spreads through sand fly bites.

### 3.4. Type of Facility First Visited

Two hundred patients consulted with various types of health care providers as the first place for seeking help with most of them visiting private health care providers.

In general, fifteen percent (15.5%) patients reported first to village doctor, 10% to the pharmacy shop, and 7.5% to “Kabiraj” (traditional health provider) upon recognition of symptom. One-third (28.5%) directly went to UZHC (Upazilla Health Complex, Government Hospital) and 15% reported first to the registered doctor. The median total delay from noticing symptoms to commencement of treatment seems higher (417 days) among those patients who reported first to traditional/spiritual healers. Majority (88%) attended at SKKRC as their 2nd- or 3rd-order treatment location. The average number of the different types of health care providers that were visited till the start of definite treatment was 2.43 (median = 3). This was without taking into account the number of repeated visits made to the same health care provider. Most of the patients (86.5%) referred to the SKKRC by registered doctor for definite treatment (Table 4).

### 3.5. Distribution of Delay

#### 3.5.1. Patient Delay

The rate of presentation to health care providers in this study was 14 per 10000 person-days of symptom recognition in the community (Table 4). Patient delay ranged from 10 days to 4,765 days (13 years) with a median of 373 days (mean: 696, IQR: 138 to 900 days). Patient delay has contributed 91% of the overall total delay to treatment. However, only 32% of patients were able to report within the first six months after developing skin manifestation. The distribution of the different components of delay is summarized in Table 5.

#### 3.5.2. Health System Delay

Health system delay in our context was the time ranging from patient's first contact to any health facility to the date of commencement of definite treatment. This comprises time spent during movement between facilities, diagnosis, and time between diagnoses and start of treatment. Generally, The system delay ranged from 0 days to 54 days, with a median total health system delay of 14 days (mean: 46.48, IQR: 7 to 44 days) was reported in this study, making rate of 447 per 10000 person-days of actively seeking for diagnosis and then treatment (Table 5). Nine percent of total delay was contributed to health system delay.

### 3.6. Determinants of Delay

The median time between onset of symptoms of PKDL and presentation at the SKKRC was 13.6 months (R: 23 to 4594 days). The patients who came from outside Mymensingh district had longest duration of delay (M: 66 months); in contrast those who were within the district had the shortest delay (M: 9 months). On bivariate analysis, age, education, occupation, and residential status had significant statistical association with patient delay (*P* < 0.05).

Educational status, occupation, number of treatment providers, and first healthcare provider had a significant statistical association with system delay (*P* < 0.05) (Table 6). However, family income could not be significant to remain in any type of delay. Thus, patient's educational status (up to class five) had longer health system delay than patients who graduated.

During the discussion, the participants explained their own perception about PKDL and the risk of PKDL in the community. One male participant said “I never seen such type of skin infection and i have no idea about the disease process and name of the disease. One believed that consumption of arsenic water may play a mechanism of disease process.”

PKDL is a mysterious disease, it causes no physical problem. One female said, “Kala azar treatment is not new for me. The last one was the second attack for me. I went directly to the public hospital last time.” One female from Trishal Upazila mentioned “People are not aware of the risk of PKDL, probably because it is not fatal disease with clear symptoms and progression.”

One male said “I went to the local doctor (drug store) near the village and took some medicine but the medicine did not work. The care giver never asked me about past history of Kala-azar treatment. We didnt have the money to buy more medicine. When my condition was not improved I noticed it to the government hospital. It is often easier (convenient) to go to a private place close by than going to the government hospital, there is usually a long queue there.”

One doctor said “There is no local terminology of PKDL. So we need to address the issue to develop BCC materials. Sister from SKKRC suggested the disease may be defined as skin problem after Kala-azar treatment.” Another staff mentioned “we need more detail discussion to find out the appropriate terminology of PKDL.”

## 4. Discussion

The study highlights the uncovered duration of infectiousness of PKDL in the community that is a potential source of spread of Kala-Azar. Approaches that take this into account may convey a more appropriate early diagnosis and control of the disease.

It is observed that the number of days of patient delay is much longer among (mean: 696 days) PKDL patients in Bangladesh. The duration of unacceptable level of patient delay observed in this study is consistent with other diseases delay related study [8–14]. A substantial proportion of the total delay of treatment was attributed to patient delay, an important preventable period of infectiousness in the community caused probably by the lack of awareness and knowledge about PKDL.

In this study area, the public health facilities are the common first choice of care for PKDL patients, with more than 60% of individuals presenting to a traditional, alternate, or nonregistered doctor initially due to easy approaches and low cost, similar to other studies specially in case of tuberculosis [12]. However, considerable number of patients contacts the private practitioners (15%) as a first choice, indicating the importance of this sector. In cases, where PKDL was suspected, private provider should refer the patient to public hospital. Significant association was observed between choice of health providers (first contact) and health system delay where visit to traditional healers increased the delay of treatment [15]. Rural residence was a risk factor for late presentation and diagnosis. This may be explained by several factors, including poorer access to health care in rural areas, difference in education levels, and lack of supervision of health staff at peripheral level [15].

The unacceptable higher patient delay was possibly due to asymptomatic nature of the disease except skin changes and the fact that community had no knowledge about the nature of PKDL. Unfortunately, most of the patients had past or family history of Kala-Azar and treated with different drugs in different time period. The patients were totally unaware about the linkage between Kala-Azar and PKDL.

This is an important preventable period of infectiousness in the community caused by the failure of recognition by the patients. Changes in the skin colour are the only symptom reported by PKDL patients which did not prompt them to seek health care. It is worth mentioning that PKDL is suspected only by the skin changes with past history of Kala-Azar. It might be that more “missed” cases occur among those who present with no background history. Some patients presented with past history of PKDL treatment with different drugs among which two patients again developed skin lesion after initial disappearances and others showed no improvement even after one year (Table 3). There is no good evidence based on treatment of PKDL; even no marker of cure is available at public health facilities.

Most of the patients of PKDL in this study area did not present to health facilities early and/or if they presented, they did not receive treatment on time and thus continued to serve as reservoirs of infection. Even though the short system delay found in this study may be explained by the development of the good general health care, choice of first health care provider can prolong the system delay. We found clear associations between delay and the type of health care provider first visited by the patient. Long system delays were more frequent by the traditional practitioner and least frequent by a registered or government physician. Education on recognition of PKDL by traditional and village practitioner may help prevent long health care provider delays, as may implementation of public-private mix projects in rural areas.

Promotion of a concerted effort to increase awareness of the signs and symptoms of PKDL in the general population in the endemic area and encouraging self-referral to health services is crucial to increase the passive case detection.

The study showed that patients who were from outside Mymensingh district had much longer delay, probably due to lack of well-developed treatment facilities and non-well-concentrated different elimination programme of that area [7].

Socioeconomic indicators are the strong determinants of the health seeking behavior of the patients which is, in turn, the main determinant of patient delay; therefore in-depth analysis was crucial to provide detailed information about the situation. Study showed that PKDL patients are a disadvantaged group in their communities. The illiteracy rates reported were significantly higher. The known association between poverty and PKDL or Kala-Azar was not found in the study though it has been well documented and the diseases have been labelled as a “disease of poverty.” Hence poverty reduction would contribute to reduction of the PKDL burden in endemic area [16, 17].

This study reveals that most of the patients are below 45 years (90%) which is consistent with the other study and the size of males is almost double the size of females. Rural people are usually suffering from PKDL as about 99% were from rural area [12, 15]. Ages of the studied patients are considered indicators of the progress in the treatment of the PKDL. Although the majority of households used bed net during sleep, its acceptability and efficacy against KA need to be ascertained [16]. Lack of knowledge about the involvement of humans in the transmissibility and infectious nature of the disease (98%) is a matter of concern for adoption of preventive measures against the disease. Awareness about the signs and symptoms of a disease can prompt patients to seek early treatment. Among the various factors cited earlier which determine treatment-seeking, preexisting health beliefs and perception about illness play an important role (Table 3).

Patients generally choose initial traditional healers; medicine shop or village practitioner resorted to nonprescribed medications from pharmacies. Meanwhile, they would also seek care at government health care provider. This study showed that visiting several health care providers was significantly associated with longer delay. Sociodemographic characteristics proved to be significant predictors of delay in almost all countries [18]. Private health care providers did not have strong linkages with the mainstream public health system. In addition, lack of continuing medical education contributes to poor knowledge and therefore poor ability to immediately diagnose a case. Education and collaboration with private health care providers in all countries are therefore essential to reduce treatment delay. The free services of the Kala-Azar and PKDL treatment should be more widely known to the community.

This study reveals that a large proportion of the community members have no knowledge on PKDL infection. Therefore, there is need to strengthen public awareness efforts against stigmatization with regard to Kala-Azar and PKDL infections.

## Figures and Tables

**Table 1:** Formula for calculating specific rates (different components of delay).

Rate	*e* (numerator)	*d* (denominator)
Total delay	Number of PKDL treatments	Total person-days of initial presentation in a community
Patient delay	Number of presentations to health provider	Total person-days of presentation before first presentation to a health provider
Health service or system delay	Number of PKDL treatments	Total person-days of skin lesion between commenced first presentation to a health provider and final treatment

**Table 2:** Baseline characteristics of the study population.

Characteristics (*n* = 200)	Categories	Number (%)	Patient delay (days)		System delay (days)	
			Mean	Median	Mean	Median
Sex	Male	129 (64.5)	761.70	417	55.74	11
	Female	71 (35.5)	576	293	29.31	13

Age (years)	01–15	63 (31.5)	542.17	290	37.62	13
	16–30	80 (25.6)	670.84	405.50	76	12
	31–45	37 (18.5)	453.76	400	16.62	11
	46–60	15 (7.5)	1171.13	814	12.13	10
	>60	5 (2.5)	446..40	344	10	5

Residential status	Urban	2 (1)	22	22	62	62
	Rural	198 (99)	702.87	380	46.33	12

Address (Upazila)	Gafargaon	80 (40)	579	251	26.06	11
	Trishal	68 (34)	674	417	40.12	12.50
	Baluka	21 (10.5)	537.40	406.31	158	11
	Mymensingh Sadar	9 (4.5)	392	344	17.11	18
	Fulbaria	5 (2.5)	317.50	317.50	25.50	25.50
	Muktagacha	4 (2)	251.30	173	13	11
	Tangail	9 (4.5)	992.67	1174	12.11	6
	Jamalpur	4 (2)	1979.50	1979.50	2	2

Educational status	Illiterate	45 (22.5)	1063	1063	16	16
	Up to 5th classes	108 (54)	769.79	420	52.49	12
	Up to 12th classes	31 (15.5)	223.33	163	24	10
	Graduate or above	16 (8)	87.46	97	16	16

Occupation of the population	Agriculture worker	28 (14)	594.71	600	14.18	11.50
	Business	33 (16.5)	1397..45	1109	11.55	10
	Daily labor	5 (2.5)	1084.80	1600	15	13
	Housewife	31 (15.5)	361	369	42.13	13
	Student	54 (27)	537.48	294	19.13	12
	Unemployed	47 (23.5)	653.85	251	127	13

Household income per month (US$)	<100	152 (76)	630.84	387	43	11
	100–150	8 (4)	1012	89.50	165.25	46.50
	151–200	38 (19)	833.97	277	35.55	13
	>200	2 (1)	767	767	37	37

Housing	Floor and wall made by mud	195 (97.5)	705.30	387	47.19	12
	Floor and wall made by brick and tin	5 (2.5)	335.80	179	18.80	15

**Table 3:** Clinical characteristics and background history of PKDL patients.

Characteristics (*n* = 200)	Categories	Number	Percentage
Past history of Kala-Azar treatment	Yes	175	87.5
	No	25	12.5

Drug used for KA treatment	Sodium Stibogluconate	141	70.5
	Miltefosine	28	1
	Liposomal amphotericin	2	1

Past history of PKDL	Yes	11	5.5
	No	189	94.5

Drug used for PKDL treatment	Sodium stibogluconate	5	2.5
	Miltefosine	4	2
	Liposomal amphotericin	2	1

Family history of KA	Yes	77	38.5
	No	123	61.5

Types of skin lesion	Hypomelanotic	148	74
	Nodular	11	5.5
	Macular	5	2.5
	Mixed	36	18

Splenomegaly	Yes	14	7
	No	186	93

Hepatomegaly	Yes	2	1
	No	198	99

RK-39 test	Positive	194	97
	Negative	6	3

Skin biopsy for LD body	Yes	75	38.5
	No	65	32.5
	Not done	40	20

Treatment	Miltefosine	146	73
	Ambisome	38	19
	Amphotericin deoxycholate	14	7
	Stibogluconate	2	1

**Table 4:** Knowledge, attitude, and practice of the study patients about PKDL.

Items	Respondents	Number	Patients delay (in days)		System delay (in days)	
			Mean	Median	Mean	Median
Heard the name PKDL	Yes	14 (7)	499.29	97	112.14	38
	No	186 (93)	710.87	380	41.54	11.15

Knowledge about PKDL	Yes	4 (2)	393	393	29	29
	No	196 (98)	702	373	41.45	12

KA is an infectious disease, transmitted by sand fly bite	Yes	18 (9)	393	393	29	10
	No	182 (91)	692.88	373	43.46	12

Complete cure of the disease is possible	Yes	42 (21)	792	402	23	14
	No	2 (1)	680	387	12	12
	Not known	156 (78)	723	320	27	11.15

Frequency of using mosquitoes net	Always	140 (70)	696	383	46.48	12
	Often	32 (16)	732	470	57.80	14

Sleeping bed	Cot	193 (96.5)	670.86	387	47.71	12
	Floor	7 (3.5)	1390	345	12.71	14

Habit of day time sleeping	Regular	56 (28)	756	420	14.41	10.50
	Occasional	118 (59)	740.50	393	25.75	12
	Rarely	26 (13)	361	121	209	20

Insecticide spray in the house in last 1 year	Yes	174 (87)	673.33	344	49.77	12
	No	26 (13)	884	503	24.50	9

First health care provider	Graduate doctor	30 (15)	603.37	238.50	154.90	11
	Union dispensary (Govt.)	8 (4)	377.50	310	19.88	13.50
	Traditional healer	14 (7.5)	604.67	417	33.47	30
	Paramedic	19 (9.5)	661.42	344	19.95	14
	Village doctor	31 (15.5)	815.10	327	15.74	14
	Upazila health complex (UZHC)	57 (28.5)	698	393	14.5	10
	Homeopath	11 (5.5)	731.7	373	64.36	12
	Pharmacy shop	20 (10)	663.40	393.50	14.35	13
	Others	9 (4.5)	1167	600	12	9

**Table 5:** Distribution of delays and rates to an event throughout the course of health seeking and start of treatment among PKDL patients.

Type of delay	Mean (median)	Rates
Total delay	768.41 (408)	13 per 10000 person-days of skin lesion
Patient delay	696.06 (373)	14 per 10000 person-days of skin lesion
Health system delay	46.48 (14)	447 per 10000 person-days of actively seeking diagnosis

**Table 6:** Relationship between sociodemographic factors and patient delay and system delay (bivariate linear regression analysis).

Factor	Patient delay			System delay		
	*β* coefficient	(95% confidence interval)	(*P* value)	*β* coefficient	(95% confidence interval)	(*P* value)
Age	0.140	(0.091–16.548)	0.048	−0.049	(−2.460–1.192)	0.492
Sex	−0.098	(−449.269–79.479)	0.169	−0.064	(−84.931–31.675)	0.369
Education	−0.139	(−202.397–(−0.180))	0.050	0.145	(−0.993–45.429)	0.041
Occupation	−0.170	(−112.070–(−11.15))	0.017	0.174	(2.849–25.086)	0.014
Residential status	0.253	(64.518–213.598)	0.000	−0.002	(−17.216–16.674)	0.975
Monthly income	0.117	(−0.005–0.056)	0.100	−0.002	(−0.007–0.007)	0.983
First health care provider	NA			−0.169	(−71.421–(−7.090))	0.017
Number of health care providers	NA			−0.147	(−24.501–(−0.681))	0.038
